# Automatic Segmentation of Facial Regions of Interest and Stress Detection Using Machine Learning

**DOI:** 10.3390/s24010152

**Published:** 2023-12-27

**Authors:** Daniel Jaramillo-Quintanar, Jean K. Gomez-Reyes, Luis A. Morales-Hernandez, Benjamin Dominguez-Trejo, David A. Rodriguez-Medina, Irving A. Cruz-Albarran

**Affiliations:** 1 Laboratory of Artificial Vision and Thermography/Mechatronics, Faculty of Engineering, Autonomous University of Queretaro, Campus San Juan del Rio, San Juan del Rio 76807, Mexico; 2 Postgraduate Studies Division, Psychology Faculty, National Autonomous University of Mexico, Mexico City 04510, Mexico; 3 Iztapalapa Unit, Department of Sociology, Division of Social Sciences and Humanities, Metropolitan Autonomous University, Mexico City 09340, Mexico

**Keywords:** stress, machine learning, thermography, face detection, short TSST, face landmarks

## Abstract

Stress is a factor that affects many people today and is responsible for many of the causes of poor quality of life. For this reason, it is necessary to be able to determine whether a person is stressed or not. Therefore, it is necessary to develop tools that are non-invasive, innocuous, and easy to use. This paper describes a methodology for classifying stress in humans by automatically detecting facial regions of interest in thermal images using machine learning during a short Trier Social Stress Test. Five regions of interest, namely the nose, right cheek, left cheek, forehead, and chin, are automatically detected. The temperature of each of these regions is then extracted and used as input to a classifier, specifically a Support Vector Machine, which outputs three states: baseline, stressed, and relaxed. The proposal was developed and tested on thermal images of 25 participants who were subjected to a stress-inducing protocol followed by relaxation techniques. After testing the developed methodology, an accuracy of 95.4% and an error rate of 4.5% were obtained. The methodology proposed in this study allows the automatic classification of a person’s stress state based on a thermal image of the face. This represents an innovative tool applicable to specialists. Furthermore, due to its robustness, it is also suitable for online applications.

## 1. Introduction

Stress is a pervasive experience that can have negative effects on mental and physical health [1], and the increase in stress in the population in recent years has been great due to external factors such as the effects of the COVID-19 pandemic and people’s isolation and return to normal life [2,3]. Identifying and managing stress can be challenging because it is often difficult to recognize and express. In recent years, researchers have explored new methods to detect stress using different bio-signals [4]. One of the most accepted methods to measure stress in people is the use of infrared thermography [5], including the use of thermal regions of interest (ROIs) on the face [6,7,8].

While extensive databases exist for the visible spectrum, there is a lack of sufficiently large and detailed databases for the thermal spectrum of the face. A proposal for such a resource is outlined in [9], where the authors present a high-resolution thermal infrared face database to improve facial thermal analysis using machine learning (ML) methods. The work includes annotation landmarks for faces, which contribute to more robust detection. An example of emotion classification is also presented.

As presented in [10], the medical literature highlights a notable difference in absolute facial skin temperature between men and women observed in clinical studies. Examination of various anatomical areas of the face revealed that, overall, men have higher temperatures than women. Some of the areas measured include the upper lips, lower lips, chin, eye socket, and cheeks. This information is relevant to the development of your gender classification system based on thermal images. It underestimates the importance of using regions of interest (ROIs) instead of reference points, as demonstrated by [11]. Their study of heat distribution in thermal images provides relevant insight into the effectiveness of ROIs, demonstrating their ability to provide more robust results by focusing on areas known to exhibit significant temperature changes.

Thermography has received extensive and growing attention for diagnostics and monitoring [12] due to the advantages of infrared or thermal cameras in determining skin temperature [13], as it is a safe, non-invasive, and non-contact technique with broad applications in health sciences [14,15]. Psychologists have documented associations between changes in facial temperature and arousal in humans, and sociologists have focused on the potential of infrared thermography to measure dimensional aspects of emotion [5,16].

Thermal ROIs are specific areas of the face, such as the forehead, cheeks, and nose, that are targeted for thermal imaging, and these ROIs have been studied extensively [5,17,18]. These ROIs are known to exhibit temperature changes that correspond to physiological responses to emotional changes [19] and stress [8], such as changes in blood flow and sweat gland activity [5]. By analyzing these temperature changes, researchers can develop algorithms to detect and quantify stress levels in individuals, as demonstrated by [20]. They proposed a system based on a variational autoencoder (VAE) to detect anomalies in a person based on the temperature changes shown. For this purpose, they defined two categories: normal state and anomaly. The VAE identified a segment with decreased skin temperature as an abnormal region and a segment with unchanged skin temperature as a normal region. The use of thermal ROIs for stress detection holds great promise for non-invasive, real-time, and accurate monitoring of stress levels. This technology has numerous potential applications, including clinical settings for stress management and in the workplace to monitor stress levels and prevent burnout. An example is provided by the research of [21], who focused on stress detection and monitoring using a mobile phone as the primary detection module, allowing detection and analysis from home. To achieve this, they used a smartphone-compatible thermal camera and a smart band as an auxiliary system to monitor participants’ heart rate, blood pressure, and pulse. Their system was tested in a pilot study designed to mimic a home environment. However, they had to take steps to create a controlled home environment to prevent interruptions during the test. For researchers planning a similar study, it is therefore essential to make well-considered decisions that consider the specific characteristics of the thermal imaging camera in the smartphone version, among other psychological factors. An implementation of emotion detection, as presented in [9], proposes a system based on facial landmark detection, face frontalization, and analysis for estimating four emotions. Although their system achieves an accuracy of 65.75%, they suggest that its modularity allows improving this accuracy by adding different tracking or analysis methods with minimal effort, making the system easily extensible. Consequently, it is possible to measure the stress level of an individual through these physiological changes. In [22], a methodology for stress detection is presented based on a hybrid approach of stress clustering at the personal level. It uses self-reports of stress decision levels as a reference and employs a smartwatch-based stress level differentiation system. The system is able to improve its performance by correcting the false labels assigned by the ML algorithm. There are several models for measuring stress using thermography, including the work of [8], which presents a thermography-based detection system validated by clinical experts. Overall, the use of thermal ROIs for stress detection represents an innovative and promising approach to improving our understanding and management of stress. It has the potential to greatly improve our ability to detect and manage stress, leading to better mental and physical health outcomes for individuals; therefore, it is important to develop methods for automatic detection and evaluation of thermal ROIs that can provide a correct classification of an individual’s stress status.

The use of Artificial Intelligence (AI) techniques such as ML and Deep Learning (DL) has been very useful and extensively studied in recent times, allowing us to create systems capable of classifying, determining, and/or selecting different types of features within a database. By using these tools in conjunction with advanced image processing available today, work has been developed for a wide range of fields, such as facial recognition, as seen in the work of [23,24], which perform facial recognition using AI. In other cases, it has also been employed for classifications or identifications among individuals; for instance, ref. [25] conducted ethnic identification of participants through the use of DL. By using AI tools in conjunction with advanced image processing within thermal images, it is possible to develop methodologies capable of determining various factors such as emotions or stress state, as demonstrated for [8,9].

The aim of this work was to develop a methodology to automatically detect facial thermal ROIs for the evaluation and classification of three states, baseline, stress, and relaxation, using ML. The system can identify five different areas on a person’s face, namely the nose, right cheek, left cheek, forehead, and chin, with an accuracy over 95% while the individual stands in front of a thermal camera. An automatic extraction of the thermal data from each ROI is then performed to create a database. Subsequently, this database is used to create a ML model for stress state classification, resulting in a model with an accuracy of 95.4%. The developed tool is easy to use and applicable to various purposes, such as thermoregulation research, as it can be used for temperature assessments over time. It can also be used in medical environments in hospitals, among others.

The main contributions of the work are as follows:

- Intelligent methodology for automatic detection of five regions of interest: forehead, nose, right cheek, left cheek, and chin. It is based on the histogram of oriented gradients (HOG) and a supported vector machine (SVM), allowing it to achieve an accuracy of 96.66% and to be implemented online.
- Thermal analysis of the obtained regions of interest after applying a short TSST test, as well as the creation of a database with this information.
- Intelligent system based on machine learning that allows the detection of baseline, stress, or relaxation after a short TSST protocol. Its performance metrics are accuracy ≥to 91.3%, precision ≥to 92%, recall ≥to 91% and F1-score ≥to 95%, for the three states to be detected.

## 2. Materials and Methods

**Figure 1:**
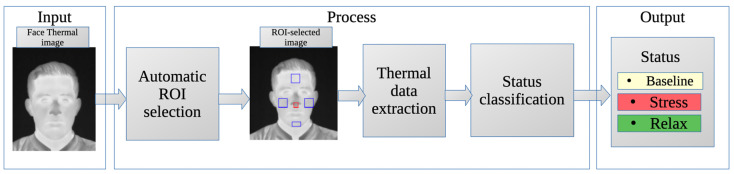
General methodology for condition classification.

The general methodology proposed for this work is shown in Figure 1. In the first step, the thermographic images used as input for the system are displayed. Then, the ROIs are automatically selected, the mean temperature values are obtained for each one, and the state of the subject is classified according to its thermal information. The result is the classification between the three possible states: baseline, stress, and relaxation.

The primary result of this study is the proposed methodology for automatic evaluation of thermal images of the human face. This methodology was developed by combining the automatic ROI selector for thermal face images, the process of obtaining average temperature values within the ROIs, and the intelligent classifier for baseline, stress, and relaxation. The method takes a thermal image of a person’s face as input and is capable of autonomously selecting the ROIs, evaluating their average temperature, and automatically classifying the person’s status in the image.

This method consists of two stages of ML The first stage involves HOG + SVM, which automatically determines the regions of interest (ROIs) in the face of a thermal image (as described in Section 2.4). Then, the temperature values of the ROIs are extracted according to the process described in Section 2.5, which serve as inputs to the second stage of ML. The second stage uses an SVM (Section 2.6) that, using the average temperature values of the ROIs as inputs, classifies the state into baseline, stress, and relax as outputs.

### 2.1. Subjects

The study included 25 participants, 9 males and 16 females, with a mean age of 20.96 years old and a standard deviation of 1.27. All participants were university students, pursuing a degree in Sports Nursing. The study complied with the General Health Law and followed the guidelines of the Helsinki Declaration. The research project was reviewed by the Bioethics Committee for Research of the Faculty of Engineering of the Autonomous University of Queretaro (UAQ), with registration number CEAIFI-132-2019-TP.

Prior to participation, each participant provided informed consent and received a confidentiality letter. In addition, participants completed a comprehensive questionnaire covering various aspects such as activities, substance use, diet, health status, medication use and, for female participants, details on the timing of the menstrual cycle.

### 2.2. Technological Equipment and Protocol

A FLIR A310 camera with a thermal sensitivity of 0.05 at 30 °C, an infrared resolution of 320 × 240 pixels, and a spectral range from 7.5 to 13 µm was used to capture the thermal images. The camera was securely mounted on a tripod at a distance of 1.2 m from the subject. To maintain consistency with previous research [26], the emissivity of the skin was set to 0.98. A Fluke 975 air quality meter was used to monitor environmental conditions. To facilitate the study, a controlled environment conducive to thermal imaging was established in a room measuring 2.5 m long, 3 m wide, and 2.5 m high. The room was maintained at a temperature of 20 ± 2 °C, with uniform lighting, no external light exposure, and a relative humidity of 45 to 60%. An air conditioner was used to maintain the desired environmental conditions.

### 2.3. Protocol Aplication

**Figure 2:**
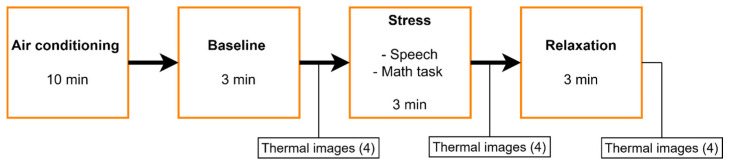
Short protocol based on the Trier Social Stress Test.

The protocol was administered by experts in the field of psychology. It consisted of four phases and was based on the Trier Social Stress Test [27,28], but in a shorter form (Figure 2). In the first phase, participants were welcomed individually into the air-conditioned room. They were instructed to sit down in the chair provided and were given a 10-min acclimatization period to adjust their body temperature and become comfortable. In the second phase, the baseline was obtained. Then, in the third stage, a stress activity was performed, during which the participants had to imagine a speech they would give to an audience and performed a math activity. Finally, in the last stage, a relaxation activity based on diaphragmatic breathing was carried out. The last three stages were performed over a period of 3 min, and four thermal images were taken at the end of each stage. Therefore, 12 images were obtained per participant. A total of 300 images were obtained for the whole population. They were asked to face the thermographic camera throughout the protocol.

### 2.4. Automatic ROI Detection

**Figure 3:**
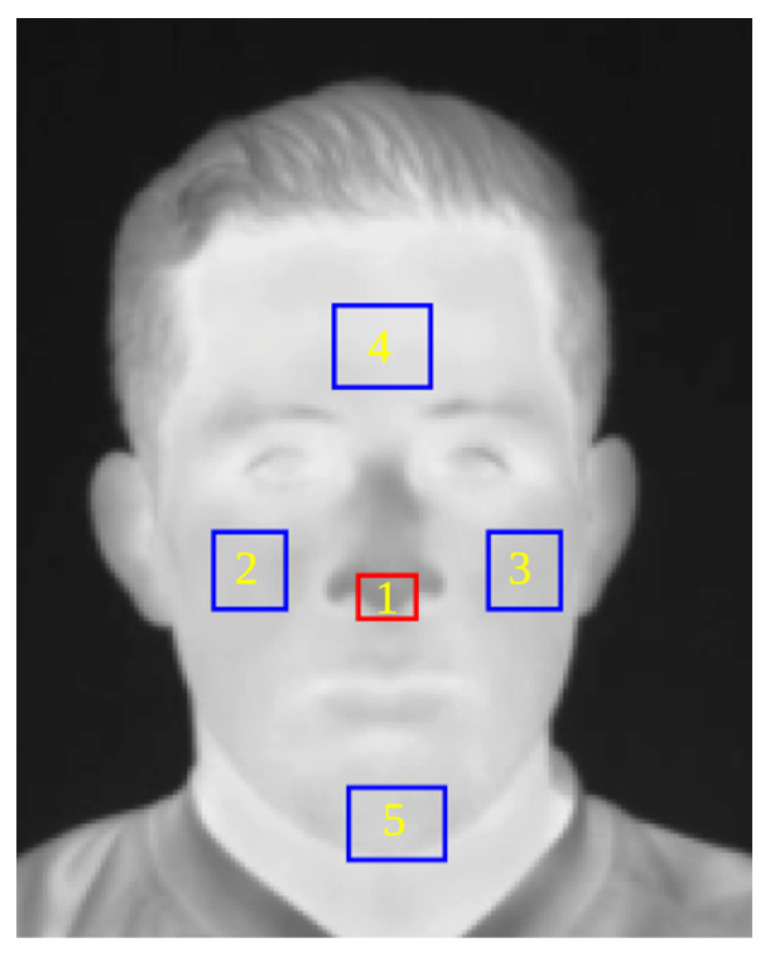
Segmented ROIs to evaluate thermal behavior.

An automatic ROI detection method was proposed, considering the ROIs according to previous studies [5]. Figure 3 shows the ROIs where temperature was evaluated, 1—nose, 2—right cheek, 3—left cheek, 4—forehead, and 5—chin.

**Figure 4:**
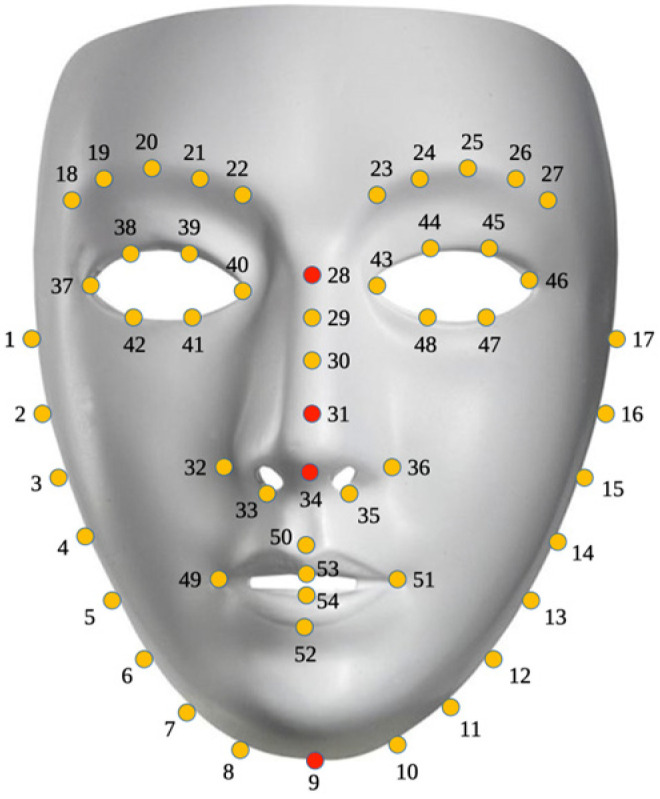
Numbered face landmarks (Numbers 9, 28, 31 and 34 highlighted in red are those considered in this work.

Once the ROIs to be analyzed are selected, it is necessary to create a system capable of automatically detecting them in a thermal image. To achieve this goal, the landmark dispersion diagram shown in Figure 4 was developed. For this phase, images from a database by [29] were used in addition to our own database containing a total of 2556 thermographic images. A total of 1980 images were used to train the system and 576 images were randomly selected to test the system. Following the steps outlined by [30], a landmark predictor using a histogram of oriented gradients (HOG) and a supported vector machine (SVM) were trained using the Python 3 software and the scikit-learn [31] and dlib [32] libraries. After training the system, thermal images with detected and annotated landmarks are obtained, as shown in Figure 3.

Only four (highlighted in red) of the landmarks obtained from the detector were used as references to locate the ROIs. Landmark number 34 was used to determine the coordinates of the nose, landmark 31 was used to determine the coordinates of both cheeks, landmark 28 was used to determine the coordinates of the forehead, and finally, landmark 9 was used to determine the coordinates of the chin. With the coordinates of the landmarks for each ROI, an equation was generated to locate the area and each pixel within each ROI in the thermal image. Equations (1)–(5) were proposed to select the pixels to be evaluated within the ROIs. Equation (1) is used for the nose ROI, Equation (2) for the right cheek ROI, Equation (3) for the left cheek ROI, Equation (4) for the forehead ROI, and Equation (5) for the chin ROI.

Nose

(1) $$\overset{SNx+\frac{w}{16}}{\underset{i=SNx-\frac{w}{16}}{\sum }}\overset{SNy}{\underset{j=SNy-\frac{h}{13}}{\sum }}Img\left[i\right]\left[j\right];$$

Right cheek

(2) $$\overset{SRx-\frac{w}{5}}{\underset{i=SRx-\frac{w}{3}}{\sum }}\overset{SRy+\frac{h}{14}}{\underset{j=SRy-\frac{h}{14}}{\sum }}Img\left[i\right]\left[j\right];$$

Left cheek

(3) $$\overset{SLx+\frac{12w}{35}}{\underset{i=SLx+\frac{w}{5}}{\sum }}\overset{SLy+\frac{h}{14}}{\underset{j=SLy-\frac{h}{14}}{\sum }}Img\left[i\right]\left[j\right];$$

Forehead

(4) $$\overset{SFx+\frac{w}{12}}{\underset{i=SFx-\frac{w}{12}}{\sum }}\overset{SFy-\frac{h}{9}}{\underset{j=SFy-\frac{h}{4}}{\sum }}Img\left[i\right]\left[j\right];$$

Chin

(5) $$\overset{SCx+\frac{w}{12}}{\underset{i=SCx-\frac{w}{12}}{\sum }}\overset{SCy}{\underset{j=SCy-\frac{h}{8}}{\sum }}Img\left[i\right]\left[j\right];$$

where SNx and SNy are the coordinates of the nose, SRx and SRy are the coordinates of the right cheek, SLx and SLy are the coordinates of the left cheek, SFx and SFy are the coordinates of the forehead, and SCx and SCy are the coordinates of the chin in the x and y axes, respectively. w represents the width and h represents the height of the detected face. Using this information, the summation of the grayscale value of the pixels at the coordinates i,j of the image (Img[i][j]) is obtained.

**Figure 5:**
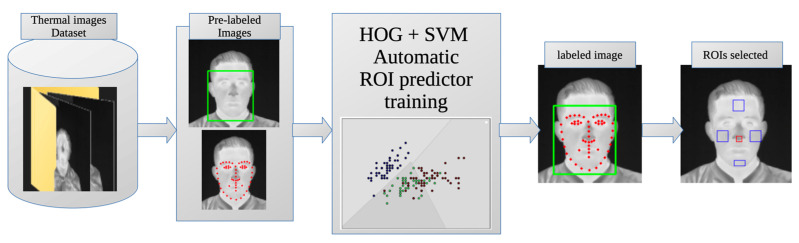
Methodology for automated ROI selection.

This leads to the development of a methodology capable of automatically detecting the ROIs in a thermal image of a person’s face. The overall diagram of this methodology is shown in Figure 5, and the output is a thermal image with the selected ROIs, as illustrated in Figure 3.

### 2.5. Thermal Data

**Figure 6:**
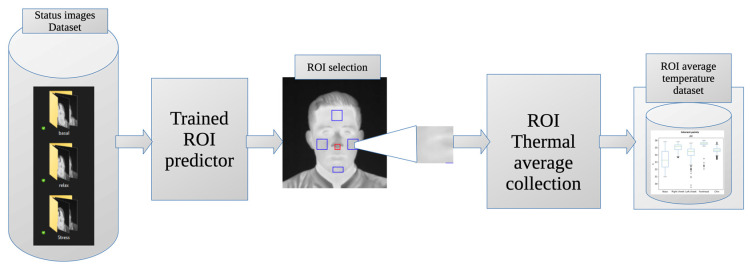
Thermal data extraction methodology.

The method for automatically detecting ROIs in a thermal image of the face (Section 2.4) was used to develop a new method for automatically extracting the mean temperature values within these ROIs (Figure 3). For this study, we used a thermal database obtained during the application of the protocol (Figure 2), consisting of a total of 300 thermal images of 25 participants (12 images per person), divided into three groups: baseline, stress and relaxation. These groups correspond to the three selected states within the TSST (Figure 6).

To calculate the average temperature of each region of interest (ROI), a thermal matrix was generated from the images using Equation (6) as suggested by [33].

(6) $$T_{r}=T_{min}+\left(\frac{T_{gray}}{T_{mgv}}\left(T_{max}-T_{min}\right)\right)$$

where *T_r_* is the thermal value of the selected pixel in the thermogram. In essence, *T_r_* represents the temperature information encoded in that pixel. The variables *T_max_* and *T_min_* refer to the minimum and maximum temperature values in degrees Celsius and define the temperature range covered by the thermal data in the image. *T_gray_* is the grayscale value of the pixel under consideration. Since the thermal image is in grayscale, the value of *T_gray_* reflects the grayscale value at the specific pixel and falls within a range from 0 to 255. In addition, *T_mgv_* represents the largest grayscale value found anywhere in the entire thermogram.

Once the temperature values of each pixel within each ROI are obtained, an average value is calculated, and a new database is created with the average temperature values of the ROIs in each image. This new database will be used for training the classifier proposed in Section 2.6.

### 2.6. Smart Status Classification

**Figure 7:**
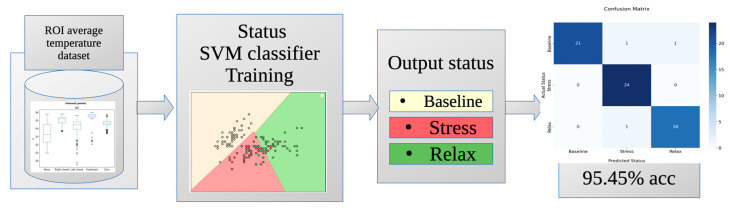
Smart status classifier chart.

Using a database of average temperature values for the ROIs from previously categorized images, we proposed a ML classification system for baseline, stress, and relaxation states (Figure 7). The dataset consists of 300 images from 25 participants in this study, with 240 images (from 20 participants) used for training and 60 images (from 5 participants) used for testing. The construction of this classifier involved the use of Python 3 software and the pandas and scikit-learn libraries. We used an SVM for classification, incorporating a grid search to determine optimal parameters. Ultimately, a radial basis function (rbf) kernel with a C-value of 100 and a gamma of 10 was selected. To validate and test the classifier, a k-fold cross-validation with k = 5 was performed.

## 3. Results

This section presents the main results of the proposed methodology. First, the results of the automatic segmentation of the ROIs are shown. Then, the results of the thermal analysis of the ROIs are reported and, finally, the results of the classification stage are summarized.

### 3.1. Automatic ROI Selection

**Figure 8:**
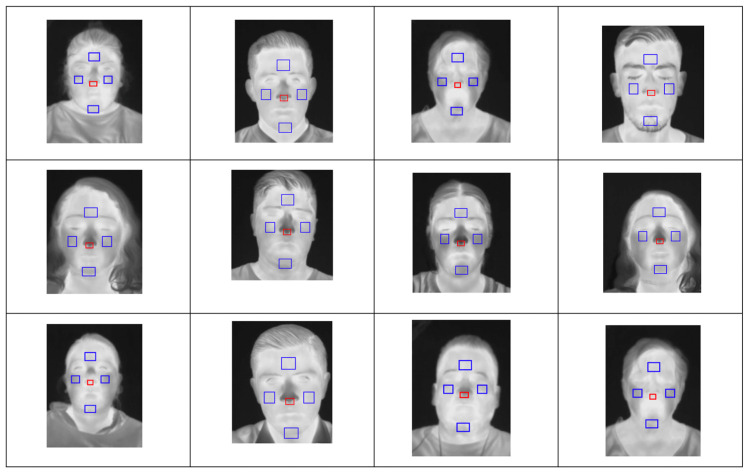
Face ROI detection results matrix.

A methodology was developed to automatically detect five facial ROIs: nose, forehead, right cheek, left cheek, and chin. To test the system, the data partition described in Section 2.4 was used, training the system with 1980 images, and testing it with 576 images, resulting in an accuracy of 96.66%. Once the system for automatic detection of regions of interest was created, trained, and tested, it was used to evaluate thermal images of the face from the database specifically collected for the purposes of this study. The selection, based on direct inspection, was performed with 100% effectiveness, due to the fact that all images were taken facing the camera. Figure 8 shows a random selection of thermal images with ROIs automatically selected by the proposed system.

### 3.2. ROIs Temperature Extraction

A database with the average temperature values of each ROI was obtained (Table 1). It is important to mention that the temperature values are presented in °C. This information is stored and analyzed for later use in the training of the final system. A Friedman statistical test was performed to determine the statistical significance of the data, which was significant in three of the five ROIs (*p* < 0.05).

**Figure 9:**
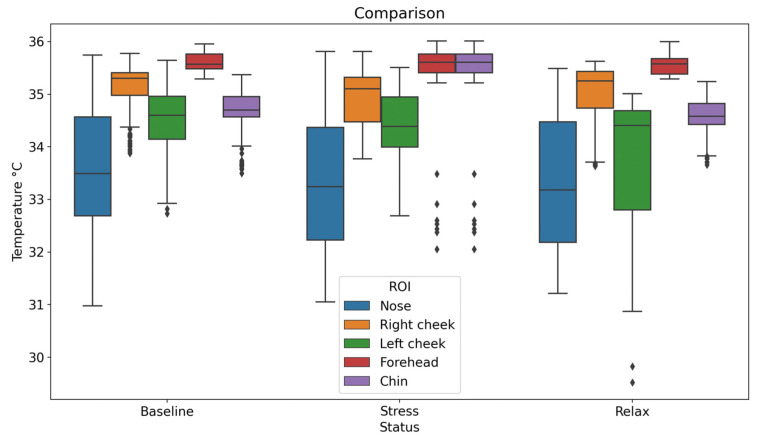
Thermal data distribution in the ROIs.

Table 1 shows the mean temperature and standard deviation (SD) of the participants in each selected ROI in the three different conditions: baseline, stress, and relaxation. This table is used to compare how temperature values vary across different facial ROIs and conditions; according to the values shown in the SD columns, we can infer that the data are not too widely distributed. For a better visualization of the data distribution, a box-and-whisker plot of the obtained data is presented (Figure 9) [20,21].

According to the results of previous studies, the nose is known to be one of the areas that shows the most thermal changes or variations during stress and relaxation [19]. This can also be seen in the data obtained in this study, which shows significant changes in other ROIs, such as the forehead and chin.

### 3.3. Intelligent Stress Status Classifier

**Figure 10:**
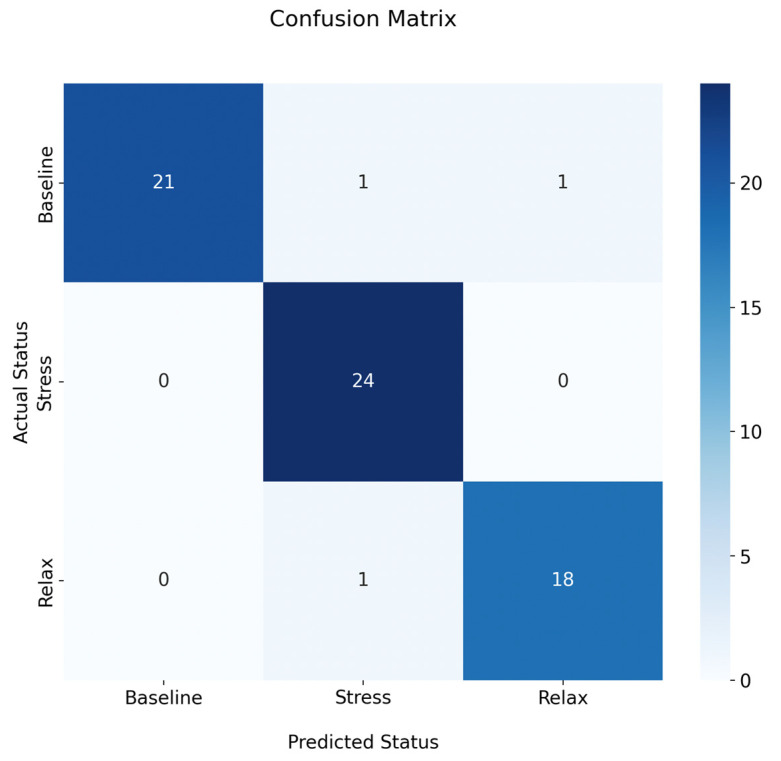
Confusion matrix for the proposed classifier.

Once the thermal database was available, it was used to create and train an intelligent classifier as described in Section 2.6. The resulting model is able to discriminate between the specific stress states with an accuracy of 95.45% and an error rate of 4.5%. Figure 10 shows the corresponding confusion matrix for the generated classifier.

The confusion matrix shows that the classifier made only three errors out of the 66 images used for evaluation. This indicates a high reliability of the classifier. Table 2 shows the evaluation metrics obtained by this classifier.

As shown in Table 2, the accuracy of the “baseline” state is 91.30%, which is the lowest of the three, but still above 90%. It accurately predicts instances of this state and has no false positives. However, it may miss some actual “baseline” instances (false negatives). Nevertheless, the F1-score of 95% suggests a commendable overall performance in classifying the “baseline” state. Regarding the “relax” state, it achieves a flawless accuracy of 100%, indicating no missed “relax” predictions. Conversely, it is correct 92% of the time. While there may be some false positive predictions for this class, there are no occurrences of false negatives. In addition, the F1-score of 96% indicates excellent overall performance. For the state “stress”, the model achieves an accuracy of 94.73%, correctly predicting instances 95% of the time. Some true “stress” instances may be missed (false negatives), but the F1-score suggests commendable overall performance in classifying “stress”. The classification model demonstrates proficiency across all three classes, with F1-score consistently above 95%, indicating a balanced trade-off between precision and recall.

Although all the work has been conducted carefully and according to best practices, the size of the database used could be an important factor to consider. For this reason, the ML-SVM technique was chosen instead of a DL approach, which would require larger input data [34]. A K-fold cross-validation was performed to ensure greater reliability in the results, which yielded encouraging and reliable results.

## 4. Discussion

The study of facial ROIs in humans has been of great interest to researchers in recent years, as knowing the average temperature values in these areas makes it possible to assess and determine thermal values related to various factors, such as stress or emotion. However, the development of automatic methods is necessary, as manual methods can sometimes bias the information. Therefore, the main contribution of this work is an automatic methodology for the detection of facial ROIs and its application in the assessment of their thermal behavior for the classification of states such as baseline, stress, and relaxation. Table 3 shows some of the most outstanding work in this area.

As shown in Table 3, extensive research has been conducted on this topic, yielding significant and valuable results. For example, ref. [35] conducted a study using the You Only Look Once (YOLO) technique and achieved a mean average accuracy of 97% in automatically detecting regions of interest (ROIs) in thermal images. However, this study did not extend to the classification of emotions associated with stress. In contrast, ref. [21] showed that changes in facial temperature were associated with stress in 71% of participants, and ref. [36] demonstrated that it could classify between baseline and high arousal and valence levels with an accuracy of 80%. Some studies have aimed to determine the specific emotion displayed by participants, achieving accuracies of 65.75% [9] and 84.72% [24]. However, these studies do not allow us to determine whether participants are in a state of stress or relaxation. Knowing this information would allow for a correlation between the type of emotion and the state of stress. In their research, ref. [22] used AI algorithms to assess stress levels using questionnaires and a smartwatch, achieving 100% accuracy in high-level calculations. However, the standard accuracy achieved by the classifier was 81.82%, even when qualitative inputs were included. On the other hand, ref. [19] demonstrated a method capable of determining the emotion displayed by a participant by evaluating facial ROIs in thermal images using a top-down hierarchical classifier, achieving 89.9%. Furthermore, ref. [8] reported a stress state classification accuracy of 91% in people using ROIs on the face and fingertips as input, using rule-based methods and heuristic knowledge. As a result, this study developed an automated methodology using ML for ROI detection and stress level assessment, achieving an accuracy of 95.4%. This allows for automatic classifications in real time, as shown in Figure 10 and Table 2, confirming that the classifier consistently provides reliable results in determining whether a participant is stressed. The ability to assess and classify a person’s stress state simply by obtaining a thermal image of their face has several potential applications, including providing instant stress diagnostics to assist psychologists during their sessions.

Despite the good results obtained, the proposal has some limitations, such as the need to correctly acquire the thermal images of the face, since a blurred, non-frontal or obstructed image limits the correct selection of ROIs and the acquisition of the mean temperature, which could bias the results obtained. Therefore, it is necessary to take care of the positioning for the thermal imaging and to respect the conditions mentioned in Section 2.2. Also, although we have a significant number of participants, it would be crucial to include a larger number of participants and therefore a larger number of thermographic images, which could lead to better results. On the other hand, it would be essential to cross-check the results obtained with other types of markers or psychological tests to validate the result, since this method is based only on temperature changes, which could be due to other factors not related to stress or, even more, to external factors. Finally, it would be useful to carry out tests with different types of AI algorithms.

As future work, it is considered to extend the database to improve the reliability of the classifiers, as well as to conduct a study involving a comparative analysis of ML techniques for the detection of regions of interest (ROIs) and the classification of stress status. In addition, with a more enriched dataset, the adoption of new classification techniques, such as the incorporation of DL techniques, is proposed. This, in conjunction with a large database, could potentially yield improved results. On the other hand, it is also suggested for future work to perform an analysis of the effect of perturbations on the detection of ROIs, since, for example, a participant may constantly move his face, which could cause a poor detection of ROIs and bias the classification obtained.

## 5. Conclusions

This study presents an automatic methodology for detecting regions of interest (ROIs) and performing thermal assessments to classify three states: baseline, stress, and relaxation. The implementation of this approach has facilitated the creation of an online system capable of assessing an individual’s stress state with an accuracy of over 95%, achieved through the analysis of facial thermal images. Specifically, the detection of facial ROIs, including the nose, forehead, right cheek, left cheek, and chin, was based on ML techniques incorporating algorithms such as HOG and SVM, resulting in a test accuracy of 96.66%. Then, to evaluate the model with another database, thermographic images were taken during the application of the short TSST protocol. Following the detection process, a database was created documenting the temperatures of the identified ROIs. This dataset was then used to train and validate a ML classifier (SVM) capable of identifying the stress state of the subject, distinguishing between baseline, stress, and relaxation with an accuracy of 95.54%, which is approximately 5% better than the most similar previous work. In particular, the robustness of this research allows its extrapolation to other applications requiring the evaluation of thermal behavior over time. The adaptability of the methodology allows it to operate in real time, such as during the monitoring process carried out by a thermographic camera. An obvious application lies in the assessment of thermoregulation, addressing the need to understand the organism’s ability to regulate temperature effectively.

## Figures and Tables

**Table 1:** Thermal data average and standard deviation in the ROIs.

	Basal		Stress		Relax		Friedman Test	
ROI	Mean	SD	Mean	SD	Mean	SD	*p*	Q
Nose	33.50	1.22	33.28	1.31	33.30	1.23	0.36	2.03
Right cheek	35.09	0.51	34.90	0.61	35.00	0.57	**<0.01**	**10.05**
Left cheek	34.48	0.72	34.32	0.73	33.81	1.17	**<0.01**	**11.83**
Forehead	35.60	0.18	35.42	0.76	35.57	0.18	0.40	1.81
Chin	34.65	0.44	34.68	0.52	34.56	0.33	**<0.01**	**10.00**

Where ROI is the selected region of interest, mean is the average of the temperature in the ROI and SD is the standard deviation (bold values indicate *p* < 0.05).

**Table 2:** Proposed classifier evaluation metrics.

Metric	Accuracy	Precision	Recall	F1-Score
Baseline	91.30%	100%	91%	95%
Relax	100.0%	92%	100%	96%
Stress	94.73%	95%	95%	95%

Where F1-Score is the harmonic mean of precision and recall.

**Table 3:** Comparative table of works related to stress detection using thermographic imaging.

Work	Method	Database	Highlights	Results
[35]	You Only Look Once (YOLO)	Public database, only 781 thermal images were used	− It identifies the ROI on the human face where temperatures are highest (ear, eye, forehead, and full face)	mAP = 97.00%
[21]	HAAR classifier, model 68 face landmark and HOG feature detection	Not mentioned	− The variability of temperature and heart rate as important indicators of stress − Smartphone stress thermography is a promising method for monitoring human stress	Stress-induced temperature changes occurred in 71% of participants
[36]	Genetic algorithms (Fisher LDA)	Own data, 12 participants, 9 females	− Facial thermal ROIs − Baseline condition − Classification between baseline and high arousal and valence levels	Adjusted classification accuracies around 80%
[9]	HOG-SVM; HOG features and a random forest classifier	Own database with 2936 thermal images; image size (1024 × 768 pixels)	− Automatic ROIs detection − Classification for four basic emotions (neutral, happy, sad, and surprised)	65.75% accuracy
[24]	CNN and HAAR features	FER dataset, grayscale portraits of people, image size (48 × 48 pixels)	− Facial recognition-based movie rating system that can detect and classify emotions (neutral, angry, disgusted, fearful, happy, sad, and surprised)	84.72% accuracy
[22]	HR and EDA signals, SVM, Random Forest, K-NN, PCA-LDA, and MLP	Data from all participants (32), including baseline, cognitive load, stress, and recovery sessions	− Platform-independent stress level detection system that works with off-the-shelf smartwatches and smartbands	81.82–100% accuracy
[19]	Top-down hierarchical classifier	Own data of 44 participants, 8 women, and 36 men	− Face ROI selection − Classification of five emotions − Self-calibration	89.9% accuracy
[8]	Rule-based method, heuristic knowledge with ROIs in fingers and face	Own database from 100 of which 70 were stimulated by the TSST protocol and 30 were not induced to human stress	− Automatic face ROI and finger detection − Classification of stress and no stress − Use of the TSST	91.0% accuracy
**This work**	**Automatic detection and classification of thermal ROIs using ML (SVM)**	**Proprietary database of 300 thermal images carefully obtained during the short TSST protocol**	− **Fast automatic ROI detection** − **Fully autonomous methodology** − **Carefully selected database** − **Use of ML**	**95.45% accuracy**

Where mAP means; mean average precision.

## Data Availability

The database may be shared upon request and with the permission of the authors.
